# Ring Stripping, Ring Cutting, and Growth Regulators Promote Phase Change and Early Flowering in Pear Seedlings

**DOI:** 10.3390/plants12162933

**Published:** 2023-08-14

**Authors:** Xiaojie Zhang, Yueju Wu, Xiaoming Wang, Wenfang Wang, Mingxia Huang, Zitan Ma, Jianying Peng

**Affiliations:** College of Horticulture, Hebei Agricultural University, Baoding 071000, China; zhangxiaojietyy@163.com (X.Z.);

**Keywords:** pear, phase change, growth regulators, ring stripping, ring cutting

## Abstract

Hybrid breeding is the most important means of selecting pear (*Pyrus*) varieties, but a long juvenile period severely restricts the selection of new varieties. In this study, we used ‘Yuluxiang’ × ‘Akituki’ 4-year-old seedling trees to study the effects of plant growth regulators, ring stripping, and ring cutting on the promotion of phase change and flowering to assist in shortening the breeding cycle. A single application of 100 mg/kg 6-BA + 1000 mg/kg PP333 was most effective in promoting phase change and flowering. This treatment effectively inhibited the growth and thickening of annual shoots, significantly increased soluble sugar and protein contents in buds, increased the ABA content by 45.41%, decreased the IAA content by 7.35%, increased the expression of the flower-promoting genes *FT* and *LFY* by 2273.41% and 1153.71%, respectively, and decreased the expression of the flower-suppressing gene *TFL1* by 74.92%. The flowering plant rate increased by 23.34% compared to the control. Both ring stripping and ring cutting were effective in promoting phase change and flowering, significantly increasing the flowering rate, inflorescence number, and the number of flowering plants. For improving the flowering rate, the ring-stripping treatment had the strongest effect and effectively inhibited the growth and thickening of annual shoots, while also significantly increasing the soluble sugar and protein contents in buds, reducing the contents of IAA and GA3 by 8.73% and 50.12%, respectively, increasing the expression of *FT* and *LFY* by 80.01% and 821.14%, respectively, and reducing the expression of the flower-suppressing gene *TFL1* by 59.22%. In conclusion, ring stripping, ring cutting, and spraying of 100 mg/kg 6-BA + 1000 mg/kg PP333 were effective in promoting phase change and early flowering in seedling trees.

## 1. Introduction

Plant growth and development involve three periods: juvenile, phase change, and mature. The phase change period is when a plant transitions from the juvenile to mature phase. Plants at the juvenile stage do not possess the ability to flower [1]. In the phase change period, plants transition from a juvenile to mature state and from nutritional to reproductive growth, undergoing a series of morphological and physicochemical changes [2,3] before acquiring the ability to flower, undergo bud differentiation, flower, and fruit [4]. The time taken for fruit trees to complete the phase change determines the time required for flowering and fruiting. The selection and breeding of new pear varieties primarily rely on a cross-breeding pathway, but the juvenile period of pears is long, comprising 4–9 years, and it severely restricts the selection and breeding of new varieties. Promoting pear seedling tree phase change to promote early flowering and fruiting, is key to shortening the pear breeding cycle [5].

Studies have shown that seedling fruit trees must experience a minimum amount of growth and accumulation of nutrient reserves to enter the transformation stage. Stage transition in fruit trees is regulated by endogenous and exogenous signals, including light, temperature, hormones, and nutrition. Growth regulators applied as exogenous sprays, along with ring-stripping or ring-cutting treatments, can regulate hormone levels and nutrient accumulation in buds. Plant hormones play various roles in the regulation of flower buds; for example, in pear, Jas (Jasmonic acid) and ethylene play negative regulatory roles in the phase transition, whereas ABA (Abscisic Acid) and IAA (Indole-3-acetic Acid) play positive regulatory roles [6]. Liu used 5-year-old pear seedling trees as test materials to study the effect of spraying plant growth regulators on flower formation, and found that the mixed spraying of 6-BA (6-Benzylaminopurine) and ethephon could promote flower bud differentiation of pear seedling trees [7]. Wang applied five growth regulators to biennial chestnut seedlings and selected the best measures to promote flowering [8]. In rice, mutations in *OsAOS1*, a key gene for JAs biosynthesis, led to lower JAs content and early flowering [9]. In apples, the growth hormone content is higher in the mature stage than in the juvenile stage [10]. The plant stage transition is a complex process involving a series of regulatory genes. Corbusier showed that *FT* genes in *Arabidopsis* are expressed in leaves and encode *FT* proteins that are transported to the stem end and activate expression of floral meristematic tissue genes in complexes with FD proteins, thereby promoting flowering [11]. The overexpression of *TFL1* can prolong flowering in plants with delayed flowering [12]. *LFY* plays an important role in promoting plant flowering and the protein product is an important transcription factor regulating flowering that has a low expression in the nutritional growth stage [13]. *LFY* expression can promote expression of genes characteristic of flowering organs, and *LFY* can link upstream flowering-time genes in distinct flower-development regulatory pathways. Downstream genes related to *LFY* can link upstream flowering-time genes and downstream homozygous heterozygous genes related to flowering in distinct flower-development regulatory pathways to achieve the regulation of plant flowering [14,15].

So far, there is a lack of a practical and ideal method to promote the early flowering of pear hybrid offspring. In this study, annual shoot development, nutrient content in buds, and endogenous hormone content were analyzed following the spraying of different combinations of growth regulators as well as ring-stripping and ring-cutting treatments, and the results were validated by q-PCR assay. Exploring the promotion effects of different methods on the stage transformation of pear seedlings, a regulatory model was proposed for the optimal combination of growth regulators and ring-stripping and ring-cutting treatments to promote the stage transformation of pear seedlings. The aim is to obtain practical and effective methods to shorten the breeding period, accelerate the breeding process, and provide guidance for the early and high yield of young fruit trees.

## 2. Materials and Methods

### 2.1. Plant Materials

F1 progenies of 4-year-old ‘Yuluxiang’ × ‘Akituki’ were grown at a plant spacing of 0.5 m × 3.0 m.

### 2.2. Experimental Design

#### 2.2.1. Plant Growth Regulator Treatment

Ten ‘Yuluxiang’ × ‘ Akituki ’ progenies with consistent growth were selected in each group, with three biological replicates and seven treatments for a total of 210 plants. Trees were sprayed with plant growth regulators as a single spraying treatment (24 May) or two spraying treatments (24 May and 8 June), with two treatments of water as the control (Table 1). Growth regulators used in the experiment: KT30 (Forchlorfenuron); 6-BA (6-Benzylaminopurine); Eth (Ethephon); PP333 (Paclobutrazol). The selection of a combination and concentration of growth regulators was based on previous results [8,16].

Three shoots from each tree were randomly selected and tagged, and their length and thickness were recorded.

Buds were picked on 30 June 2021 and stored at −80 °C. The soluble sugar and protein contents and endogenous hormone content were determined, and real-time fluorescence quantitative PCR was conducted.

On 20 December 2021, the length and thickness of the marked shoots were measured following the cessation of growth, and the growth and thickening of the shoots was calculated.

On 7 April 2022, the plant stage transition and flowering status were investigated.

#### 2.2.2. Ring-Stripping and Ring-Cutting Treatments

10 ‘Yuluxiang’ × ‘Akituki’ progenies with consistent growth were selected in each group, with 3 biological replicates and 7 treatments for a total of 90 plants. Ring-stripping and ring-cutting treatments were applied, with one ring-stripping treatment on 24 May and two ring-cutting treatments on 24 May (10 cm above ground) and 8 June (15 cm above ground), along with a blank control. Measurements were taken as per Section 2.2.1.

### 2.3. Analytical Methods

For the determination of bud soluble sugar content, the anthrone colorimetric method was used [17]. The bud soluble protein content was determined by the Komas Brilliant Blue method [18]. For determination of the bud endogenous hormone content, ZR (trans-Zeatin-riboside), IAA, ABA, and GA3 (Gibberellin A3) levels in buds were determined using an enzyme-linked immunosorbent assay [19].

For real-time fluorescence quantitative PCR, the test apparatus for grinding the samples was sterilized in an autoclave and transferred into an oven for drying. Samples were pre-cooled in liquid nitrogen and removed after the liquid nitrogen stopped boiling. The samples selected for this study were buds of pear trees, and such plant samples contain polysaccharide polyphenols. An RNAprep Pure Polysaccharide Polyphenols Total Plant RNA Extraction Kit (TIANGEN BIOTECH (BEIJING) Co., Ltd., Beijing, China) was used according to the manufacturer’s instructions. The extracted RNA was tested for concentration and quality, and reverse transcription was performed to synthesize the first strand of cDNA. An EasyScript One-Step gDNA Removal and cDNA Synthesis SuperMix kit (TIANGEN BIOTECH (BEIJING) Co., Ltd., Beijing, China) was used for total RNA reverse transcription according to the manufacturer’s instructions. Real-time fluorescence quantitative PCR was used to determine the spatiotemporal expression of the flowering-related genes *FT*, *LFY,* and *TFL1*, using the pear actin gene as an internal reference. The cloned pear *FT*, *LFY*, *TFL1*, and actin sequences were used as templates to design fluorescent quantitative primers using Premier 5.0 (Premier Biosoft, San Francisco, CA, USA), gene expression was analyzed using a CFX Connect Optics Module Burroughs fluorescent quantitative PCR instrument, and the relative expression of the genes was calculated using 2^−ΔΔCt^. The primer sequences are shown in Table 2.

### 2.4. Statistical Analysis

Data were organized using Excel 2015 (Microsoft, Redmond, WA, USA), graphs were created using Origin 2018 (Originlab, Northampton, MA, USA), and statistical analysis was performed using SPSS 26 (IBM, Armonk, NY, USA).

## 3. Results

### 3.1. Effect of Plant Growth Regulators on Phase Change and Flowering

#### 3.1.1. Effect of Plant Growth Regulators on Shoot Growth

**Figure 1:**
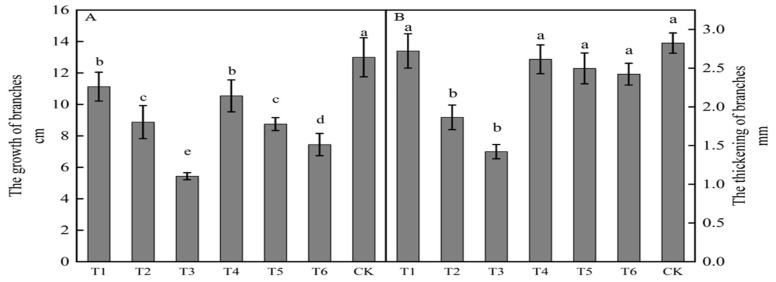
Effects of growth regulators on new bud growth and thickening. The numbering of the horizontal axis is the same as Table 1. (**A**)The growth of branches. (**B**) The thickening of branches. Different lowercase letters represent significant differences at *p* < 0.05.

The growth of new shoots in ‘Yuluxiang’ × ‘Akituki’ progenies was inhibited by spraying growth regulators (Figure 1A), being significantly less following all six treatments than in the control. Compared with the results for the control, each treatment inhibited growth, with reductions of 7.57, 5.55, 4.25, 4.13, 2.45, and 1.87 cm for T3, T6, T5, T2, T4, and T1, respectively. All six active treatments had less shoot thickening than that in the control, but only results for T2 and T3 were significance. Compared with the control, treatments T3, T2, T6, T5, T4, and T1 reduced thickening by 1.40, 0.96, 0.40, 0.33, 0.21, and 0.10 mm, respectively, with T3 being the most effective in inhibiting new growth and thickening.

#### 3.1.2. Effect of Plant Growth Regulators on Bud Nutrient Content

**Figure 2:**
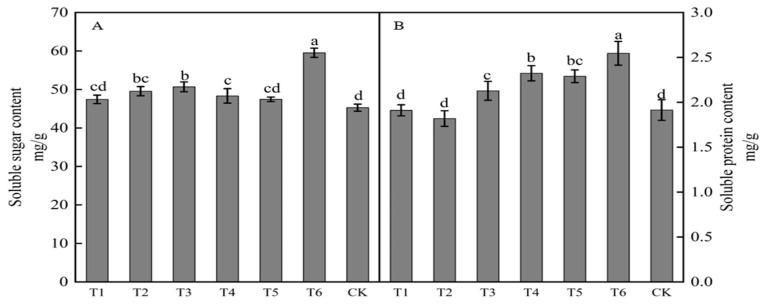
Effects of growth regulators on bud soluble sugar and soluble protein content. The numbering of the horizontal axis is the same as Table 1. (**A**) Soluble sugar content. (**B**) Soluble protein content. Different lowercase letters represent significant differences at *p* < 0.05.

The spraying of growth regulators affected the soluble sugar content of buds (Figure 2A). The soluble sugar content in buds from highest to lowest with different treatments was in the order T6 > T3 > T2 > T4 > T5 > T1 > CK. Results for treatments T6, T3, T2, and T4 were significantly higher than those in the control, with increases of 31.51, 11.93, 9.44, and 6.75%, respectively. The spraying of growth regulators also affected the soluble protein content of buds (Figure 2B). The soluble protein content following treatments T1 and T2 was lower than that in the control, whereas the soluble protein content with the other four treatments was higher than that in the control. The soluble protein content in buds from highest to lowest with different treatments was in the order T6 > T4 > T5 > T3 > CK > T1 > T2. Treatments T6, T4, T5, and T3 were significantly higher than that in the control, with increases of 33.00, 21.39, 19.64, and 11.15%, respectively. Treatments T3, T4, and T6 significantly increased both the soluble sugar and protein contents in buds.

#### 3.1.3. Effect of Plant Growth Regulators on Bud Endogenous Hormone Content

**Figure 3:**
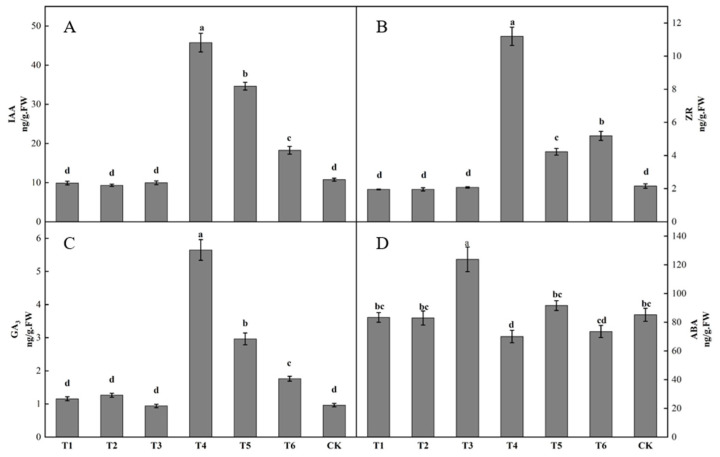
Effect of growth regulators on endogenous hormone content in buds. The numbering of the horizontal axis is the same as Table 1. (**A**) IAA content. (**B**) ZR content. (**C**) GA3 content. (**D**) ABA content. Different lowercase letters represent significant differences at *p* < 0.05.

Differences in bud endogenous hormone content was observed after spraying different plant growth regulators (Figure 3). The IAA content in buds following the T4 treatment was the highest, being significantly higher than that following the other treatments. The IAA content in buds following the T2 treatment was the lowest. The IAA contents in buds following the T1, T2, and T3 treatments were lower than that in the control by 7.87, 13.32, and 7.35%, respectively. The IAA contents in buds following the T4, T5, and T6 treatments were all higher than that in the control, increasing by 325.94, 222.24, and 69.92%, respectively (Figure 3A). The ZR content in buds following the T4 treatment was significantly higher than that following the other treatments. The ZR contents in buds following the T1, T2, and T3 treatments were all lower than that in the control, decreasing by 9.31, 9.18, and 3.63%. The ZR content in buds following the T4, T5 and T6 treatments were all higher than that in the control, increasing by 419.27, 96.06, and 140.55%, respectively (Figure 3B). The T1, T2, T4, T5, and T6 groups all had higher GA3 contents in buds than the control, increasing by 19.99, 31.25, 485.91, 207.36, and 82.74%, respectively. The T3 treatment had a lower GA3 content in buds than the control, decreasing by 2.40% (Figure 3C). The T3 group had the highest ABA content in buds, being significantly higher than those of the other groups. The ABA content in buds following the T4 treatment was the lowest. The ABA contents in buds following the T1, T2, T4, and T6 treatments were all lower than that in the control, by 2.11, 2.56, 17.75, and 13.61%, respectively. The ABA contents in buds following the T3 and T5 treatments were both higher than that in the control, by 45.41 and 7.62%, respectively (Figure 3D).

**Figure 4:**
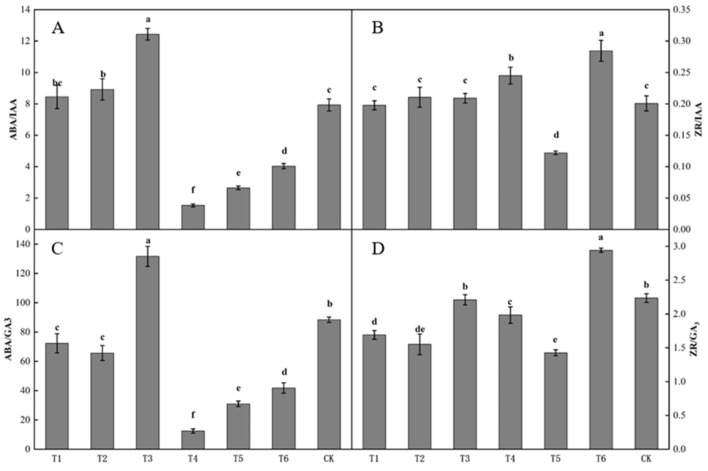
Effect of growth regulators on the ratio of endogenous hormones in buds. The numbering of the horizontal axis is the same as Table 1. (**A**) ABA/IAA. (**B**) ZR/IAA. (**C**) ABA/GA3. (**D**) ZR/GA3. Different lowercase letters represent significant differences at *p* < 0.05.

The ratio of endogenous hormones in buds differed significantly after spraying with different plant growth regulators (Figure 4). The ratio of ABA to IAA in buds following the T3 treatment was the highest, being significantly higher than that following the other treatments. The ratio of ABA to IAA in buds following the T4 treatment was the lowest (Figure 4A). The ratio of ZR to IAA in buds following the T6 treatment was the highest, being significantly higher than that following the other treatments (Figure 4B). The ratio of ABA to GA3 in buds following the T3 treatment was the highest, being significantly higher than that following the other treatments. The ratio of ABA to GA3 in buds following the T1, T2, T4, T5, and T6 treatments was significantly lower than that in the control. The ratio of ABA to GA3 in buds following the T4 treatment was the lowest, being significantly lower than that following the other treatments (Figure 4C). The ratio of ZR to GA3 in buds following the T6 treatment was significantly lower than that in the control. The ratio of ZR to GA3 in buds following the T3 and CK treatments was significantly higher than those following the T1, T2, T4, and T5 treatments, and the ratio of ZR to GA3 in buds following the T5 treatment was the lowest (Figure 4D).

#### 3.1.4. Effect of Plant Growth Regulators on the Expression of Flowering-Related Genes

**Figure 5:**
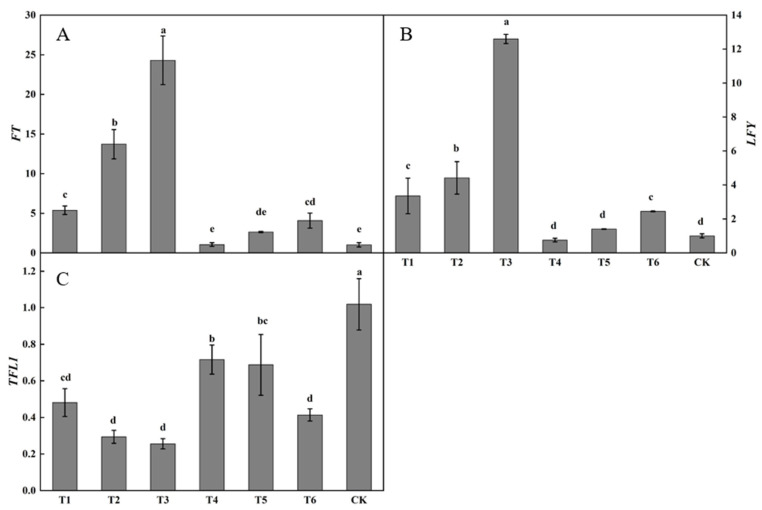
Effects of growth regulators on the expression of *FT*, *LFY*, *TFL1* in buds. The numbering of the horizontal axis is the same as Table 1. (**A**) The expression of *FT*. (**B**) The expression of *LFY*. (**C**) The expression of *TFL1.* Different lowercase letters represent significant differences at *p* < 0.05.

Substantial differences were present in the relative expression of *FT* in buds after spraying plant growth regulators (Figure 5A). The relative expression of *FT* following the T3 treatment was the highest, being significantly higher than that following the other treatments. The relative expression of *FT* following the T2 treatment was significantly higher than those following the T1, T2, T4, T5, or T6 treatments or in the control. The relative expression of *FT* following the T1 treatment was significantly higher than those following the T4 or T5 treatments and in the control. The relative expression of *FT* following the T1, T2, T3, T4, T5, and T6 treatments were all higher than in the control, by 425.49, 1239.34, 2273.41, 2.77, 157.73, and 297.97%, respectively.

Considerable differences were observed in *LFY* expression in buds after spraying plant growth regulators (Figure 5B). The relative expression of *LFY* following the T3 treatment was the highest, being significantly higher than those following the other treatments. The relative expression of *LFY* following the T2 treatment was significantly higher than those following the T1, T4, T5, and T6 treatments or in the control. The relative expression of *LFY* following the T1, T2, T3, T5, and T6 treatments was higher than in the control, increasing by 233.90, 339.65, 1153.71, 39.66, and 143.34%, respectively. The relative expression of *LFY* following the T4 treatment was lower than that in the control, decreasing by 24.97%.

Large differences were present in *TFL1* expression in buds after spraying plant growth regulators (Figure 5C). The relative expression of *TFL1* in the control was the highest, being significantly higher than that following the active treatments. The relative expression of *TFL1* following the T4 and T5 treatments was not significantly changed, whereas the relative expression of *TFL1* following the T4 treatment was significantly higher than those following the T1, T2, T3, and T6 treatments. The relative expression of *TFL1* following the T1, T2, T3, T4, T5, and T6 treatments was lower than that in the control by 52.78, 71.17, 74.92, 29.69, 32.53, and 59.42%, respectively.

The expression of the flower-promoting genes *FT* and *LFY* was the highest and the expression of the flower-suppressing gene *TFL1* was the lowest following the T3 treatment.

#### 3.1.5. Effect of Plant Growth Regulators on Flowering

The spraying of different plant growth regulators had different effects on flowering (Table 3). T3 treatment gave the highest flowering plant rate of 46.67%, significantly higher than the control rate of 23.34%. The T1, T2, T5, and T6 treatments had higher results than in the control by 10.34, 14.60, 3.34, and 12.38%, respectively. The T4 treatment was slightly lower than the control, by 3.33%. In terms of average inflorescence number, the T2, T3, and T6 treatments were significantly higher than those of the T1, T4, and T5 treatments or the control, whereas the T1, T2, T3, and T6 treatments significantly increased the average inflorescence number compared with the results for the control. The highest mean number of inflorescences was recorded for the T3 treatment with 21.45. In terms of average total flower formation, the T2, T3 and T6 treatments were significantly higher than those of the T1, T4, and T5 treatments and the control. The T1, T2, T3, T5, and T6 treatments significantly increased the total flower formation compared with that in the control. The T3 treatment had the highest average total number of flowers with 139.10. In terms of the juvenile span, the T3 and T6 treatments had results significantly lower than those with the T1, T5 treatments and in the control, and the T3 and T6 treatments significantly shortened the juvenile span compared with that results in the control. The T6 treatment had the shortest juvenile span of 192.33 cm.

### 3.2. Effect of Ring Stripping and Ring Cutting on Phase Change and Flowering of Hybrid Progenies

#### 3.2.1. Effect of Ring Stripping and Ring Cutting on Shoot Growth

**Figure 6:**
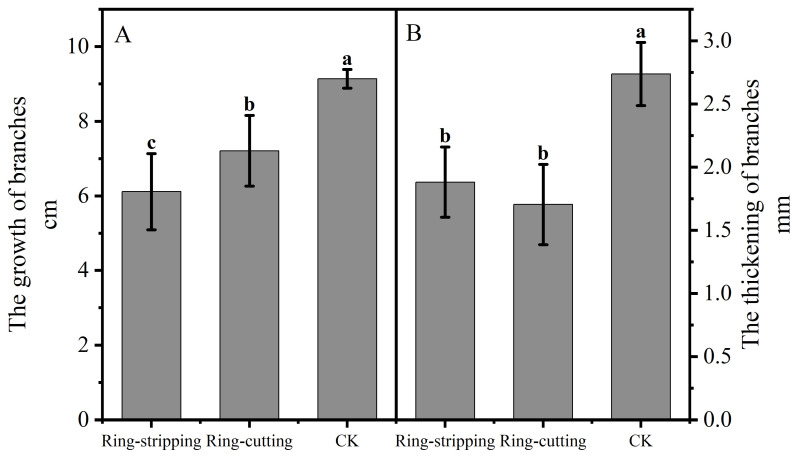
Effects of ring-stripping and ring-cutting on new buds growth and thickening. (**A**) The growth of branches. (**B**) The thickening of branches. Different lowercase letters represent significant differences at *p* < 0.05.

Shoot growth was significantly lower following both the ring-stripping and ring-cutting treatments compared with the results in the control, with 3.02 cm less growth observed following the ring-stripping treatment and 1.93 cm less following the ring-cutting treatment (Figure 6A). Branch thickening was significantly lower following both the ring stripping and ring-cutting treatments compared with results in the control, with 0.86 mm less thickness following the ring-stripping treatment and 1.03 mm less thickness following the ring-cutting treatment (Figure 6B). The ring-stripping treatment had the greatest effect in inhibiting branch growth and thickening.

#### 3.2.2. Effect of Ring Stripping and Ring Cutting on Bud Nutrient Content

**Figure 7:**
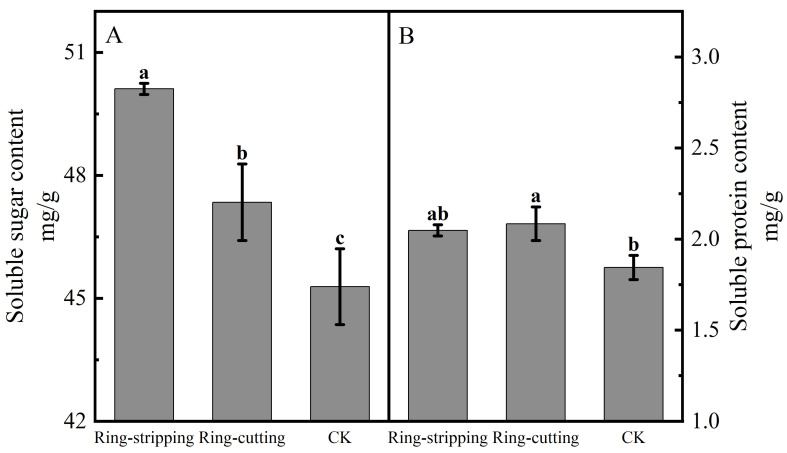
Effects of ring-stripping and ring-cutting on bud soluble sugar and soluble protein content. (**A**) The bud soluble content. (**B**) The bud soluble protein content. Different lowercase letters represent significant differences at *p* < 0.05.

Both ring stripping and ring cutting significantly increased the bud soluble sugar content (Figure 7A). The bud soluble sugar content following the ring-stripping treatment was significantly higher than that following ring cutting, with the bud soluble sugar content following the ring-stripping treatment being significantly higher than that in the control by 10.67%, whereas that following the ring-cutting treatment was significantly higher than that in the control by 4.56%. The ring-stripping and ring-cutting treatments had different effects on bud soluble protein content (Figure 7B). The ring-cutting treatment significantly increasing bud soluble protein content by 13.10%. No significant difference in results was present between the ring-stripping treatment and the control in terms of bud soluble protein content. The ring-stripping treatment was more effective than the ring-cutting treatment in increasing the soluble sugar content of buds, whereas the ring-cutting treatment was more effective than the ring-stripping treatment in increasing the soluble protein content of buds.

#### 3.2.3. Effect of Ring Stripping and Ring Cutting on Bud Endogenous Hormone Content

**Figure 8:**
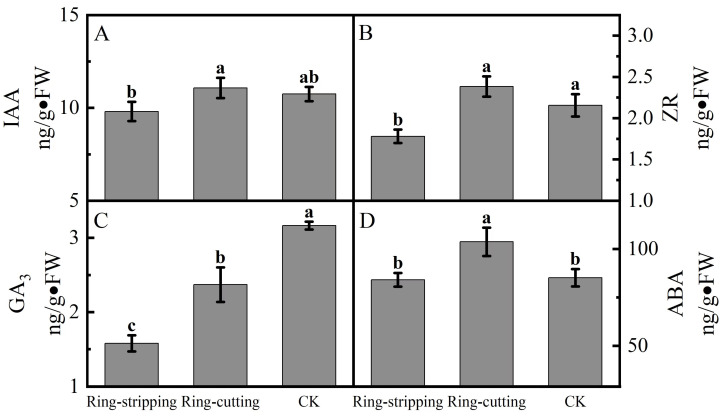
Effects of ring-stripping and ring-cutting on endogenous hormone content in buds. (**A**) IAA content. (**B**) ZR content. (**C**) GA3 content. (**D**) ABA content. Different lowercase letters represent significant differences at *p* < 0.05.

A few differences were observed in the bud endogenous hormone content among treatments. The IAA content in buds following the ring-cutting treatment was significantly higher than that following the ring-stripping treatment. There was no significant difference in the IAA content in the buds of both ring-stripping and ring-cutting treatments compared to the control (Figure 8A). The ZR content in buds following the ring-cutting treatment and in the control was significantly higher than that following the ring-stripping treatment. The ZR content in buds following the ring-cutting treatment was higher by 10.60% than the results obtained in the control, and the ZR content in buds following the ring-stripping treatment was lower by 17.43% than results for the control (Figure 8B). The GA3 contents in buds following the ring-stripping and ring-cutting treatments were significantly lower than that in the control. The GA3 content in buds following the ring-stripping treatment was the lowest, being 50.12% lower than that in the control. The GA3 content in buds following the ring-cutting treatment was 25.09% lower than that in the control (Figure 8C). The ABA content in buds following the ring-cutting treatment was significantly higher than results following the ring-stripping treatment or in the control. The ABA content in buds following the ring-cutting treatment was higher by 21.81% than that in the control. The ABA content in buds following the ring-stripping treatment was not significantly different from that in the control, although it was reduced by 1.26% (Figure 8D).

**Figure 9:**
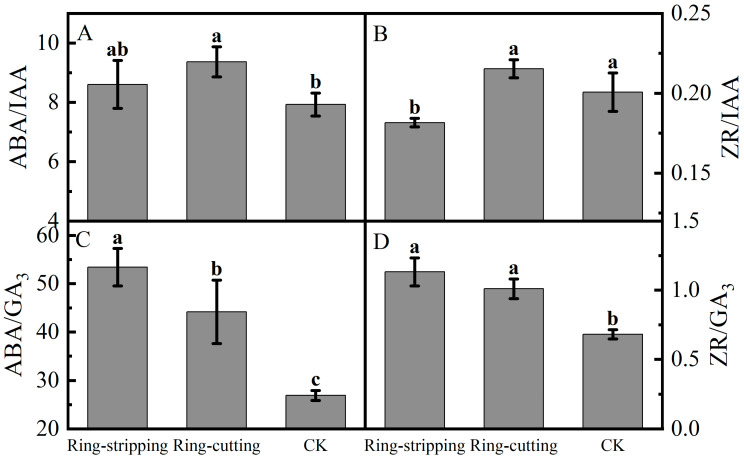
Effects of ring-stripping and ring-cutting on the ratio of endogenous hormones in buds. (**A**) ABA/IAA. (**B**) ZR/IAA. (**C**) ABA/GA3. (**D**) ZR/GA3. Different lowercase letters represent significant differences at *p* < 0.05.

A few differences were present in the ratios of bud endogenous hormone levels among the treatments. The ABA-to-IAA ratio in buds following the ring-cutting treatment was significantly higher than that in the control, increasing by 18.16%. The ABA-to-IAA ratio in buds following the ring-stripping treatment was not significantly different from that in the control, although it increased by 8.51% (Figure 9A). The ZR-to-IAA in buds following the ring-cutting treatments and in the control was significantly higher than that following the ring-stripping treatment. The ZR-to-IAA ratio in buds following the ring-cutting treatment was not significantly different from that in the control, although it increased by 7.30%. The ring-stripping treatment had resulting values that were 9.53% lower than those in the control (Figure 9B). The ABA-to-GA3 ratios in buds following both the ring-stripping and ring-cutting treatments were significantly higher than that in the control. The ABA-to-GA3 ratio following the ring-stripping treatment was the highest, increasing by 98.59% compared with that in the control. The ring-cutting treatment had a value that was higher by 64.16% than that in the control (Figure 9C). The ZR-to-GA3 ratio was significantly higher in buds following both the ring-stripping and ring-cutting treatments than in the control. The ring-stripping treatment had the highest ratio of ZR to GA3, with an increase of 66.20% compared with the resulting values for the control. The ring-cutting treatment showed an increase of 48.32% compared with the resulting values for the control (Figure 9D).

#### 3.2.4. Effects of Ring Stripping and Ring Cutting on the Expression of Flowering-Related Genes

**Figure 10:**
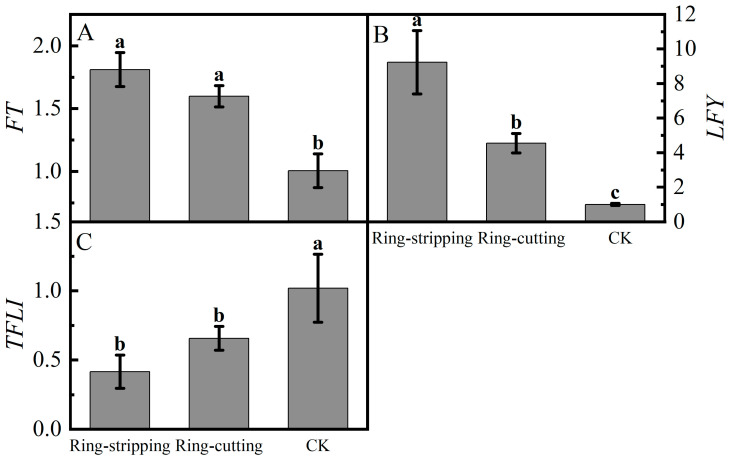
Effects of ring-stripping and ring-cutting on the expression of *FT*, *LFY*, *TFL1* in buds. (**A**) The expression of *FT*. (**B**) The expression of *LFY*. (**C**) The expression of *TFL1.* Different lowercase letters represent significant differences at *p* < 0.05.

Large differences were present in the relative expression of *FT* in buds following the ring-stripping and ring-cutting treatments (Figure 10A). The relative expression of *FT* in buds following the two treatments was significantly higher than that in the control. The relative expression of *FT* following the ring-stripping treatment was the highest, increasing by 80.01% compared with the resulting values for the control. Substantial differences were observed in the relative expression of *LFY* in buds following the ring-stripping and ring-cutting treatments (Figure 10B). The relative expression of *LFY* following the ring-stripping treatment was significantly higher than that following ring-cutting treatment and in the control, with an increase of 821.14% compared with the resulting values in the control. The relative expression of *LFY* following the ring-cutting treatment was 353.85% higher than that in the control. A considerable difference was present in the relative expression of *TFL1* in buds following the ring-stripping and ring-cutting treatments (Figure 10C). The relative expression of *TFL1* following the ring-stripping and ring-cutting treatments was significantly lower than that in the control. The relative expression of *TFL1* following the ring-stripping treatment was the lowest, being 59.22% lower than that in the control. The relative expression of *TFL1* following the ring-cutting treatment was 35.58% lower than that in the control. Both the ring-stripping and ring-cutting treatments significantly increased the relative expression of *FT* and *LFY* and decreased the relative expression of *TFL1*, with the ring-stripping treatment being more effective.

#### 3.2.5. Effect of Ring Stripping and Ring Cutting on Flowering

The ring-stripping and ring-cutting treatments had different effects on the flowering of seedling trees (Table 4). In terms of the flowering rate, the ring-stripping and ring-cutting treatments had results that were significantly higher than the results obtained for the control, with the ring-stripping treatment having the highest flowering plant rate at 47.84%. In terms of the average number of inflorescences, both the ring-stripping and ring-cutting treatments had results that were significantly higher than that in the control, with the ring-stripping treatment giving the highest number at 16.33. In terms of the number of flowers, both the ring-stripping and ring-cutting treatments gave results that were significantly higher than that in the control, with the ring-cutting treatment having the highest number at 101.25. The ring-stripping and ring-cutting treatments had results that were significantly lower than that in the control in terms of the juvenile span, with the ring-stripping treatment being the lowest at 190.25 cm. Both the ring-stripping and ring-cutting treatments significantly increased the rate of flowering plants, number of inflorescences, and total number of flowering plants and significantly decreased the juvenile span.

## 4. Discussion

Fruit tree breeding is largely based on hybrid breeding methods, but the long juvenile period of seedling fruit trees considerably restricts progress. Hence, promoting phase change and early flowering is important in seedling fruit trees for breeding. Adequate nutrient reserves and growth form the basis for the phase change in seedling fruit trees, with nutrient content and shoot growth having important effects on flowering. Sugars, as the main assimilated substances in plants [20,21], along with proteins, form the material basis for cell division and differentiation [22], and both substances play an important role in flower bud differentiation [23,24]. Spraying PBO on young apple trees of ‘Changfu 2′ significantly inhibited new growth and promoted early flowering [25]. Spraying PP333 on ‘New Red Star’ apple seedlings inhibited new growth, shortened the internode length, and enabled the production of numerous axillary flower buds, thus accelerating the transition from nutritional to reproductive growth [26]. In this study, T3-treated ‘Yuluxiang’ × ‘Akituki’ seedling progenies showed the effective inhibition of annual shoot growth by 7.57 cm and shoot thickening by 1.40 mm compared with result in the control. The soluble sugar and protein contents in buds increased significantly by 11.93 and 11.15%, respectively, compared with levels in the control. Spray application of 100 mg/kg 6-BA + 1000 mg/kg PP333 facilitated the accumulation of nutrients in buds [27]. The annual shoot growth and thickening in the hybrid seedlings treated using ring stripping and ring cutting were significantly lower than those in the control, and the soluble sugar and protein contents in buds were higher than those in the control; thus, ring-stripping and ring-cutting treatments also facilitated the accumulation of nutrients in buds [28].

Plant flowering must be preceded by floral bud differentiation, which represents a process of cellular differentiation involving complex physiological and biochemical changes [29,30]. Numerous studies have shown that regulation by endogenous plant hormones is related to flower bud formation, with the balance between the content of various plant hormones playing a decisive role in flower bud differentiation. ZR is involved in flower organ morphogenesis and promotes cell division and growth. In *Lycium barbarum*, high levels of ZR promoted flower bud differentiation [31]. In apples, high levels of ABA and IAA favored the induction of flower formation and floral organ primordial differentiation [32], but high levels of GA3 were detrimental to floral bud differentiation [33]. The ABA content following the T3 treatment was significantly higher than that in the control, but levels of IAA, ZR, and GA3 were not significantly different to those in the control. However, the ratios of ABA to IAA and ABA to GA3 were significantly higher than those in the control. In terms of the flowering plant rate, T3 showed a value of 46.67%, significantly higher than that in the control at 23.33%. The GA3 content following the ring-stripping treatment in ‘Yuluxiang’ × ‘Akituki’ progenies was significantly lower than that in the control, and the ratios of ABA to GA3 and ZR to GA3 were significantly higher than those in the control, although the ZR content was significantly lower than that in the control. The ZR and ABA contents following the ring-cutting treatment were significantly higher than those in the control, whereas the GA3 content was significantly lower than that in the control. Even though the IAA content was not significantly different to that in the control, the ratios of ABA to IAA and ZR to IAA were significantly higher than those in the control, and the flowering plant rates of the seedling trees after ring stripping and ring cutting were significantly higher than that in the control. The T3, ring-stripping, and ring-cutting treatments all promoted a phase change in pear trees and significantly increased the rate of flowering, but were not controlled by a single hormone, rather showing multiple hormones acting in synergy to regulate phase change and flowering [34].

At the genetic level, it is possible to provide a better explanation for the effects of growth regulators, ring stripping, and ring cutting on phase change in seedlings [35]. The overexpression of *FT* from citrus and tomato in *Arabidopsis* resulted in early flowering [36,37]. *LFY* overexpression in *Arabidopsis*, tobacco, citrus, and poplar can promote flowering transition and lead to earlier flowering [15,38]. *TFL1* overexpression in *Arabidopsis* appeared to delay flowering and reproductive growth [39]. Gyllenstrand showed that the *TFL1* gene can be used to regulate the length of the juvenile stage in fruit trees [40]. The high expression of *FT* and *LFY* and low expression of *TFL1* can promote completion of the stage transition in seedling trees. Among the six active treatments with growth regulators used in this study, the T3 treatment (100 mg/kg 6-BA + 1000 mg/kg PP333) increased the expression of *FT* and *LFY* and suppressed the expression of *TFL1* in buds, with the resulting effect being the strongest increasing the expression of the flower-promoting genes *FT* and *LFY* by 2273.41 and 1153.71%, respectively, while decreasing the expression of the flower-suppressing gene *TFL1* by 74.92%. Similarly, ring-stripping and ring-cutting treatments increased the expression of *FT* and *LFY* in buds and suppressed the expression of *TFL1*, which significantly increased the rate of flowering and promoted the completion of phase change in pear trees.

This study screened the optimal treatment for promoting phase change and early flowering by spraying different combinations and concentrations of plant growth regulators on 4-year-old hybrid offspring. It indicates that after a certain amount of nutritional growth, the hybrid offspring enter the transformation period and are sprayed with appropriate plant growth regulators, which will promote stage transformation and early flowering. Ring stripping and ring cutting treatments have the same effect. The research results have broad application prospects in promoting the transformation of pear seedlings and early flowering, as well as promoting the early fruit and high yield of young trees.

## 5. Conclusions

Both the spraying of plant growth regulators and ring-stripping and ring-cutting treatments significantly increased the expression of the flower-promoting genes *FT* and *LFY* and decreased the expression of the flower-suppressing gene *TFL1*. Nutrients, endogenous hormones, and the expression of flowering-related genes interact, jointly regulating the phase change and early flowering in pear. Both ring stripping and ring cutting, as well as the spraying of 100 mg/kg 6-BA + 1000 mg/kg PP333, significantly promoted phase change and early flowering in ‘Yuluxiang’ × ‘Akituki’ hybrid seedlings. The ring-stripping treatment had the highest flowering rate but spraying growth regulators was more efficient and might be chosen in particular situations.

**Figure 11:**
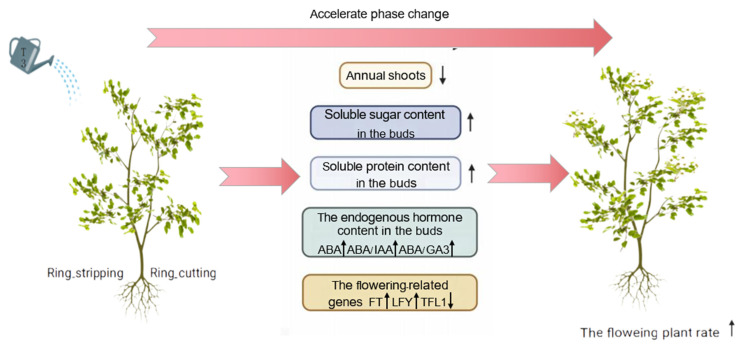
Model of the regulation of phase change in pear by exogenous T3, ring stripping and ring cutting.

Based on the research results, a regulatory model was proposed for the optimal combination of growth regulators, and ring-stripping and ring-cutting treatments to promote the phase change of pear seedlings (Figure 11).

It is worth noting that spraying plant growth regulators, and ring-stripping and ring-cutting treatments promote the phase change and early flowering of hybrid offspring. It is necessary to wait for the hybrid offspring to enter the transformation phase before it has any effect. Therefore, accurately determining the time when the hybrid offspring enters the transformation phase is crucial and also one of the directions for future research.

## Figures and Tables

**Table 1:** Combination and times of plant growth regulators sprayed on leaf surface.

Treatment	Combination of Agents	Number of Sprays
T1	1 mg/kg KT30 + 1000 mg/kg PP333	1 time
T2	50 mg/kg6-BA + 1000 mg/kg PP333	1 time
T3	100 mg/kg6-BA + 1000 mg/kg PP333	1 time
T4	1 mg/kg KT30 + 1000 mg/kg Eth	2 times
T5	50 mg/kg6-BA + 1000 mg/kg Eth	2 times
T6	100 mg/kg6-BA + 1000 mg/kg Eth	2 times
CK	water	2 times

**Table 2:** qPCR primer table.

Gene	PCR Product Length	Primer Sequence (5’→3’)	Usage
*FT*	220	F:AGCCCAAGTGACCCCAACCT R:CGGCGAAGTCTCTGGTATTGAAG	RT-qPCR
*LFY*	226	F:AGGGAGCACCCGTTCATCGT R:GCCGCATCTTTGGCTTGTTG	RT-qPCR
*TFL1*	164	F: CCTTCACTCCAACAACGCAC R: CAGGACAATCTGGGTCCGTC	RT-qPCR
*Actin*	200	F:TTGGTATGGGTCAGAAGG R: CTGTGAGCAGAACTGGGTG	RT-qPCR

**Table 3:** Effect of growth regulators on flowering conditions. Different lowercase letters represent significant differences at *p* < 0.05.

Treatments	Flowering Plant Rate/%	Average Number of Inflorescences/pcs	Average Total Number of Flowers/pcs	Juvenile Span/cm
T1	33.67 ± 6.34 b	11.67 ± 4.53 b	65.35 ± 20.88 b	230.25 ± 14.27 b
T2	37.93 ± 5.32 b	17.89 ± 3.79 a	105.55 ± 29.45 a	216.67 ± 15.06 bc
T3	46.67 ± 3.57 a	21.45 ± 4.36 a	139.10 ± 27.92 a	199.54 ± 17.74 c
T4	20.00 ± 8.35 c	4.25 ± 4.12 cd	23.80 ± 11.57 d	253.33 ± 12.08 a
T5	26.67 ± 5.73 c	6.25 ± 4.23 c	41.88 ± 10.73 c	237.67 ± 10.10 b
T6	35.71 ± 7.21 b	18.06 ± 3.82 a	112.33 ± 30.80 a	192.33 ± 18.98 c
CK	23.33 ± 5.63 c	4.50 ± 3.64 cd	23.40 ± 10.32 d	235.75 ± 11.83 b

**Table 4:** Effects of ring-stripping and ring-cutting on flowering conditions. Different lowercase letters represent significant differences at *p* < 0.05.

Treatments	Flowering Plant Rate/%	Average Number of Inflorescences /pcs	Average Total Number of Flowers /pcs	Juvenile Span /cm
Ring-stripping	47.84 ± 8.47 a	16.33 ± 4.24 a	91.45 ± 12.36 a	190.25 ± 30.21 b
Ring-cutting	43.33 ± 7.38 a	15.45 ± 5.71 a	101.25 ± 10.94 a	213.33 ± 21.98 b
CK	22.22 ± 5.82 b	4.25 ± 2.72 b	27.33 ± 8.36 b	240.25 ± 16.73 a

## Data Availability

The data could be given upon reasonable request from the corresponding author.
